# invdup(8)(8q24.13q24.3)—A Complex Alteration and Its Clinical Consequences

**DOI:** 10.3390/genes15070910

**Published:** 2024-07-12

**Authors:** Rafaella Mergener, Marcela Rodrigues Nunes, Ana Kalise Böttcher, Monique Banik Siqueira, Helena Froener Peruzzo, Milene Carvalho Merola, Mariluce Riegel, Paulo Ricardo Gazzola Zen

**Affiliations:** 1 Post-Graduate Program in Pathology, Universidade Federal de Ciências da Saúde de Porto Alegre (UFCSPA), Porto Alegre 90050-170, RS, Brazil; 2 Medical Genetics Resident, Irmandade da Santa Casa de Misericórdia de Porto Alegre (ISCMPA), Porto Alegre 90020-090, RS, Brazil; 3 Undergraduate Program in Biomedical Science, Universidade Federal de Ciências da Saúde de Porto Alegre (UFCSPA), Porto Alegre 90050-170, RS, Brazil; 4 Undergraduate Program in Biomedical Sciences, Universidade do Vale do Rio dos Sinos (UNISINOS), São Leopoldo 93022-750, RS, Brazil; nique.stranger@gmail.com; 5 Casa dos Raros, Center for Comprehensive Care and Training in Rare Diseases, Porto Alegre 90610-261, RS, Brazil; 6 National Institute of Population Medical Genetics (INAGEMP), Porto Alegre 90035-903, RS, Brazil; 7 Medical Genetics, Department of Clinical Medicine, Universidade Federal de Ciências da Saúde de Porto Alegre(UFCSPA), Porto Alegre 90020-090, RS, Brazil; 8 Irmandade da Santa Casa de Misericórdia de Porto Alegre (ISCMPA), Porto Alegre 90050-170, RS, Brazil

**Keywords:** inversion, duplication, chromosome 8, cleft lip/palate, neuropsychomotor development delay

## Abstract

Structural variation is a source of genetic variation that, in some cases, may trigger pathogenicity. Here, we describe two cases, a mother and son, with the same partial inverted duplication of the long arm of chromosome 8 [invdup(8)(q24.21q24.21)] of 17.18 Mb, showing different clinical manifestations: microcephaly, dorsal hypertrichosis, seizures and neuropsychomotor development delay in the child, and a cleft lip/palate, down-slanted palpebral fissures and learning disabilities in the mother. The deleterious outcome, in general, is reflected by the gain or loss of genetic material. However, discrepancies among the clinical manifestations raise some concerns about the genomic configuration within the chromosome and other genetic modifiers. With that in mind, we also performed a literature review of research published in the last 20 years about the duplication of the same, or close, chromosome region, seeking the elucidation of at least some relevant clinical features.

## 1. Introduction

Structural variation (SV) is known to be the main source of genetic variation in humans. Although deletions, duplications, insertions and inversions may already configure pathogenicity, they have been shown to have a critical impact on higher-order chromatin conformation and gene functioning. A combination of more than one SV may reorganize chromatin interactions by altering the DNA segments that are involved in the establishment of three-dimensional contacts [1].

Duplications of distal 8q are not common. There are only a few reports of this in the literature, most of them ranging from 8q22-qter, with a variation in the phenotype due to the duplication size. Their common dysmorphic facial abnormalities include a broad face, prominent forehead, hypertelorism, downslanting/upslanting palpebral fissures, nasal bridge alterations, micrognathia, long/short philtrum, and low-set and large ears. Other manifestations are a low birth weight, congenital heart defects, skeletal abnormalities, developmental and growth delays, a short stature, cryptorchidism in males, hypertrichosis and the frequent occurrence of seizures [2,3]. 

Inversions of chromosome 8, on the other hand, are not so uncommon. There are several reports of this in the literature, but most of them take place on the short chromosome arm or involve both arms. This pericentric inversion [inv(8)(p23.1q22.1)] may predispose individuals to other rearrangements and lead to Recombinant 8 syndrome, also known as San Luis Valley syndrome [rec(8)dup(8q)inv(8)(p23.1q22.1)] [4]. Both, the extension of the alteration and its genomic orientation, may influence the pathology of copy number variations (CNVs) once these inversions alter the directio of the binding motifs of the CTCF proteins, transcription factors that bind to tens of thousands of genomic sites, and act as a transcriptional activator, repressor and insulator [1,5].

Here, we describe the clinical manifestations of a boy with an inverted duplication of a region of chromosome 8’s long arm [invdup(8)(8q24.13q24.3)] that was maternally inherited but showed completely different outcomes between mother and child. We also explore the incidence of cleft lip/palate in their family, and attempt to determine the relationship with this alteration; in order to better understand it, we reviewed twenty years of literature reports encompassing this region and compared their phenotype. 

## 2. Case Report

**Figure 1:**
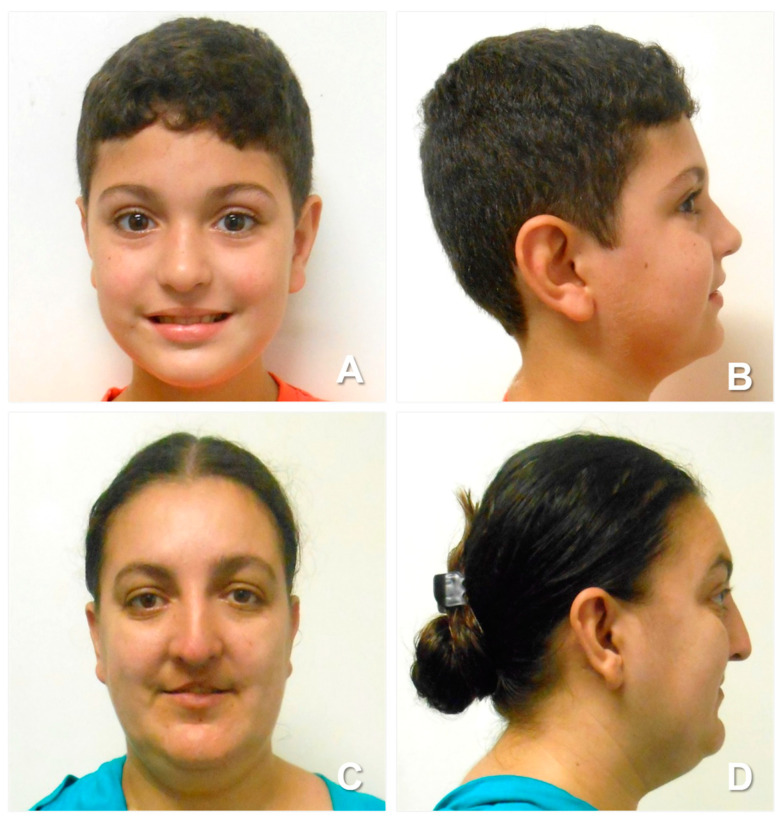
Patient and his mother. (**A**) Frontal photo of the patient. (**B**) Lateral photo of the patient. (**C**) Frontal photo of the patient’s mother. (**D**) Lateral photo of the patient’s mother.

The patient is a 14-year-old boy who underwent his first genetics evaluation at the age of 2 due to developmental delays being noticed by his family (Figure 1A,B). He is the firstborn child of a mother with a unilateral lip and palate cleft, who also reported learning difficulties at school (Figure 1C,D). The father’s medical and family history is unremarkable. 

The baby was delivered via vaginal birth, with a birthweight of 3.845 g (50th percentile), a birth height of 51 cm (50th percentile) and a head circumference of 34.5 cm (50th percentile). The Apgar score was 10 at 5 min. Regarding his development, he started walking at 1 year and 2 months, and by 2 years old, he spoke only a few words with no complete sentences. At the age of 2, a physical examination revealed a low anterior hairline, microcephaly with a head circumference of 46 cm (below 5th percentile), a hemangioma on the lower lip and dorsal hypertrichosis. The karyotyping result was 46,XY,add(8)(q24.3). 

At 3 years old, he started experiencing several episodes of seizures. A brain MRI was performed and showed normal parameters in all criteria. An electroencephalogram (EEG) was also carried out while he was awake; this presented a rare small sharp wave in the temporal region of the left hemisphere.

**Figure 2:**
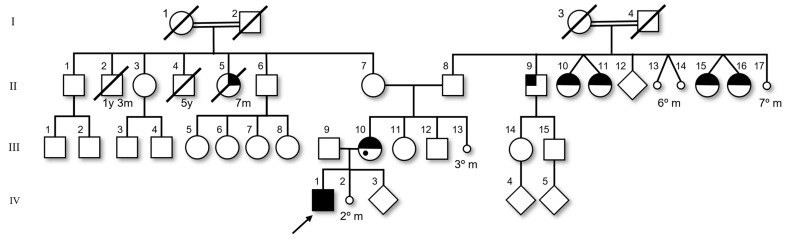
Patient’s pedigree chart. Arrow indicates the index case with neuropsychomotor development delay; Black quarter on the left: cleft lip; Black quarter on the right: cleft palate; Half black: cleft lip and palate; Black spot: learning disability. The age of death or gestacional period is indicated below each individual; y: year; m: month.

The mother also underwent cytogenetic investigation, with a karyotype result of 46,XX,add(8)(q24.3), while the maternal grandmother’s karyotype was 46,XX. It was not possible to evaluate the maternal grandfather. However, she reported other cases of cleft lip/palate and abortion in his pedigree (Figure 2). 

When the child was 5 years old, his mother became pregnant again; however, the pregnancy was terminated due to natural causes at 2 months of gestational age. They had another child with a normal karyotype two years later. 

A psychometric assessment known as the Wechsler Intelligence Scale for Children (WISC III) [6] was performed when the patient was 8 years old, and showed a full-scale IQ of 69, a verbal IQ of 71 and a performance IQ of 73. At this time, he went to school and already knew how to read. 

**Figure 3:**
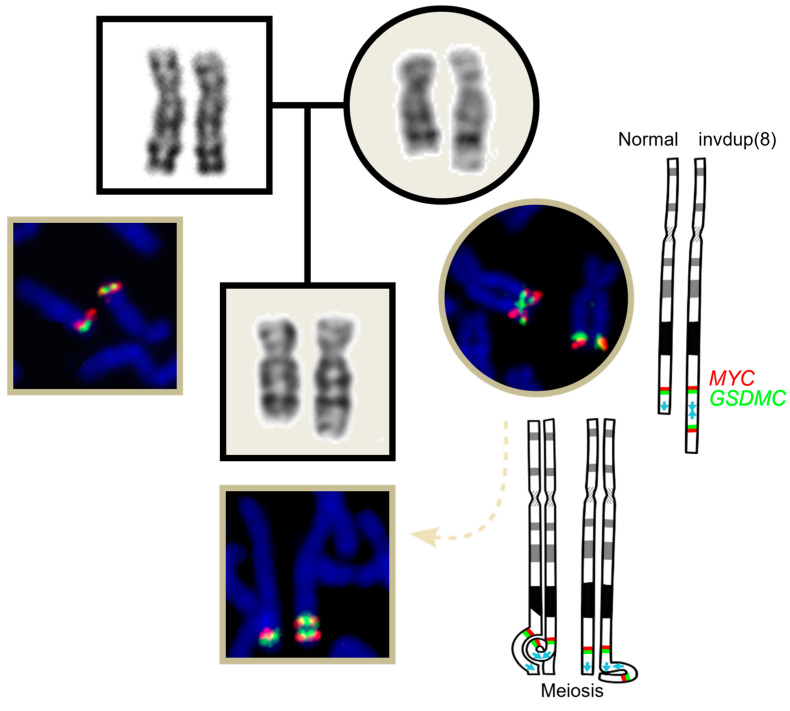
GTG banding karyotype and FISH from the index case [7] and his parents. On the right, ideograms of chromosome 8 with the probe signals and representation of the mother’s meiotic pairing. Red: probe labeling the gene *MYC*; Green: probe labeling the gene *GSDMC*; Blue arrows: representation of gene *BAI1*.

At this time, a full investigation involving fluorescence in situ hybridization (FISH) and array-based comparative genomic hybridization (array-CGH) was performed on the child, as previously described in a case series [7], and his parents (Figure 3), which allowed us to determine the orientation and length of the alteration. Thus, due to DNA isolation and oligonucleotide array-based CGH using an 8 × 60 K whole-genome platform (design 021924, Agilent Technologies, Santa Clara, CA, USA), the 17.18 Mb duplication was found in both the patient and his mother. FISH experiments were carried out through standard techniques using commercially available locus-specific probes (Cytocell, Cambridge, UK) for MYC (red) and GSDMC (green) genes, both on 8q24.21, and these showed an inverted signal pattern (red–green–green–red). Exome sequencing (ES) was unable to be performed due to patient unavailability.

According to ISCN 2020, the full cytogenetic description is given as 46,XY,der(8)(q24).ish invdup(8)(q24.21q24.21)(q24.21)(MYC+,GSDMC+)(q24.21)(GSDMC+,MYC+).arr[GRCh38] 8q24.13-q24.3(125,385,074–142,496,610) × 3 mat for the index case; 46,XX,der(8)(q24).ish invdup(8)(q24.21q24.21)(q24.21)(MYC+,GSDMC+)(q24.21)(GSDMC+,MYC+).arr[GRCh38] 8q24.13-q24.3(125,385,074–142,496,610) × 3 for his mother; and 46,XY.ish 8q24.21(MYC,GSDMC) × 2. arr (X,Y) × 1,(1–22) × 2 for his father.

His most recent consultation was at 12 years old; at the time, he no longer presented with seizures after starting valproic acid, an anticonvulsant agent, although he demonstrated agitated behavior which required the use of the antipsychotic drug Risperidone. At this point, he also underwent another psychometric assessment (WISC IV) [8], which showed a full scale IQ of 46, a verbal IQ of 48 and a performance IQ of 45. He was not adapting to regular school anymore and was sent to a governmental school called Associations of Parents and Friends of Exceptional Children (APAE—from Portuguese: “*Associações de Pais e Amigos dos Excepcionais*”), in order to receive special care.

## 3. Literature Review

We found nine cases of chromosome 8 terminal duplication from the last 20 years. Their clinical features, as well as the present case, are summarized in Table 1. Seeking a better visualization, we only kept the most frequent dysmorphic features and grouped some alterations when we found them appropriate. For a more detailed overview, see Table S1. 

**Figure 4:**
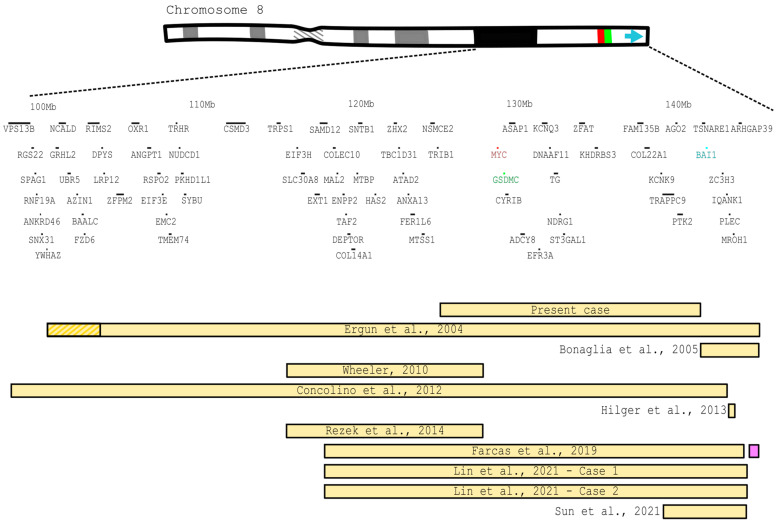
Schematic comparison of the duplicated regions of our patient and his mother with other described cases. The position and size of each gene are represented by the yellow (duplication) or purple (deletion) bars. Some of the smaller genes are not represented. A full gene description can be found in Table S1. The hatched portion of the bar marks the uncertain included region. Red: *MYC* gene, green: *GSCMC* gene and blue: *BAI1* gene [2,3,9,10,11,12,13,14,15].

The total range of the duplicated segments extends from 8q22~23→qter, and encompasses 216 genes. Some of the regions overlap, but others do not, as can be visualized in Figure 4. In order to better compare the data, all breakpoints (when available and with correctly given genome assembly) were converted to GRCh38/hg38 through Lift Genome Annotations [16]. For more detailed information about the gene analysis, see Table S2. 

From these, only Bonaglia et al., 2005 [10], reported an inverted duplication; however, the genomic coordinates were not available and, following the report, its region only overlaps ours in one gene (*BAI1*). Wheeler, 2010 [11], on the other hand, presented a case with some of the most common features, allowing phenotype/genotype comparisons among some of the characteristics. We also found one case, described by Farcas et al., 2019 [13], in which the duplication was accompanied by a minor deletion of the long arm of chromosome 8. 

Three cases had duplications caused by translocations: (1) Ergun et al., 2004 [9], reported a boy with a karyotype 46,XY, add(1)(p36).ish der(1)t(1;8)(p36.33q22.3)(wcp8,CEB108/T7+, CDC2L1+, wcp1), and through the FISH technique showed that the distal 1p monosomy was negligibly small, making the substantial duplication of chromosome 8 almost pure; (2) Rezek et al., 2014 [3], described a girl with a karyotype 46,XX,der(22)t(8;22)(q22.1; p11.1)mat., with a duplication of chromosome 8 and no pathogenic imbalances found on chromosome 22 or any other chromosomes; (3) Sun et al., 2021 [15], described a boy with 46,XY. arr 8q24.3(140,238,896–14,629,577) × 3, 21q22.3(44,491,199–48,097,372) × 1 due to the unbalanced inheritance of a paternal translocation and observed that both alterations (8q duplication and 21q deletion) were very similar regarding its clinical manifestations. 

Even though the case reported by Lin et al., 2021 [14], shows a correlation with heart defects, we found it pertinent to include it in our literature review for two reasons. The first is because its region overlaps with our case and, second, because it can illustrate the consequence of a massive copy number gain (x6–7) in this area. 

Another case that could shed light on some morphological formations regarding heart defects was described by Hilger et al., 2013 [12]; this is the smallest duplication reported, involving just three genes (*PLEC*, *EPPK1*, *PARP10*). As expected, one of the largest alterations, described by Concolino et al., 2012 [2], also displays the majority of clinical features. 

## 4. Discussion

A generally applicable rule is that phenotypes associated with deletions are more severe than those associated with duplication [17]. Here, we describe an inherited inverted duplication of chromosome 8 in two related individuals (mother and son) with distinct phenotypes that also differ from the current literature findings.

### 4.1. Facial Features

Among the facial dysmorphias, we have just a few things to disclose. The most common alterations detected in our literature review were hypertelorism, low-set ears and nasal bridge alterations, none of which were present in our case or his mother. We did not notice any genetic similarity involving hypertelorism and nasal bridge alterations, but all five authors [2,3,9,11,13] that described low-set ears reported the duplication of the *MYC* gene, with Wheeler’s patients also showing hearing loss [11]. The breakpoint of Wheeler’s patient is not completely clear as no genome assembly was specified, but the distal breakpoint is very close to the *MYC* gene. 

It is known that *MYC* family members play crucial roles in regulating cell proliferation, size, and differentiation during organogenesis and are expressed throughout inner-ear development in mice [18]. This gene may be responsible for Wheeler’s [11] patient’s hearing loss. Although it seems not to affect the outer ear, due to its role in ear formation, the presence of the *MYC* gene on the shared duplicated region of these cases may not be random. The *MYC* gene was also involved in the duplicated area in our patient and his mother, but neither he nor his mother presented any ear alterations. This outcome was also not mentioned by Lin et al., 2021 [14], who described a massive duplication in this area. This fact may illustrate the complicated interpretation of genome configuration. 

The relationship between the chromosome structure and gene expression is rather complex. It can be dynamic during processes that involve the rewiring of regulatory networks and have either very modest effects or disrupt normal gene expression, leading to pathological phenotypes [19]. Inversions can directly disrupt coding sequences or change gene expression by separating regulatory elements from the corresponding coding sequences [20,21]. 

Furthermore, as the inverted segment is an extra copy, the patient and his mother have yet one copy of the whole genomic content in the correct orientation. This may be why they do not show other clinical manifestations related to both the loss of expression and the gain of genetic material. The only report of inversion, beyond ours, was described by Bonaglia et al., 2005 [10]. Interestingly, its duplicated region starts where ours ends, which means that they do not share the same genetic burden, but this may also indicate a recombination spot in this area. 

The patient’s mother was born with a cleft lip/palate. Analyzing their pedigree chart (Figure 2), we noticed a lot of other cases of cleft lip/palate and spontaneous abortion in this family. This fact may suggest a genetic component to this condition that comes from past generations. A cleft lip and palate are the most common craniofacial human birth defects [3]. Its etiology is complex, involving genetic, epigenetic, and environmental factors. Regarding the genetic forms, it may be syndromic or not, and among the non-syndromic forms, some chromosome regions are associated, including the 8q24 [3]. This region encompasses the locus Orofacial cleft 12 (OFC12, MIM#612858), where the variant rs987525 ([GRCh38] 128933908) on a Long Non-Coding RNA (*CCDC26*) is tightly correlated with this feature [22,23]. Beyond this variant, the role of this region in the development of a cleft lip/palate remains to be clarified. 

Despite this, a cleft lip and/or palate was not seen in our index case, and was present in only two other cases in our literature review [3,13]. One of these cases, Rezek et al., 2014 [3], had a breakpoint very close to *CCDC26*, which could show that this phenotype is caused by interference in the genomic configuration. However, the case described by Wheeler, 2010 [11], who used the same genomic coordinates as Rezek et al., 2014 [3], did not have a cleft lip and/or palate. This complication may illustrate the incomplete penetrance of this condition or be just a matter of breakpoint deviation, due to array-CGH platform differences. 

One recent research study hypothesized that an explanation for the atypical clinical presentations of individuals carrying the same variant consists of the modulation of other variants in the genetic background [24]. In this scenario, the main duplication may be segregating together with another variant, responsible for cleft lip/palate, in our patient’s family or, as suggestive as the pedigree chart may be towards a genetic condition, this outcome could be simply a result of environmental factors, shared among this family.

Another dysmorphic trait observed in the patient’s mother was downslanted palpebral fissures, a characteristic of another two literature cases with no overlap in their duplicated segment [11,15]. Also, there was a report of one case with upslanted palpebral fissures [9], which makes the relationship between the genotype and phenotype regarding this feature unclear.

### 4.2. Skin Anomalies

The girl described by Wheeler, 2010 [11], displayed extensive hirsutism. Because our patient is a boy, we report it as hypertrichosis. There is a similarity in the duplicated area from 125,385,074 to 129,419,458 (4.034.384 bp) between Wheeler’s [11] case and ours, as only four genes are in this region: *TRIB1*, *LRATD2*, *POU5F1B* and *MYC*. Here, we highlight another role of *MYC*, namely hair follicle morphogenesis. *MYC* expression was found in mouse skin throughout the hair follicle cycle, promoting the proliferation of hair matrix keratinocytes as well as the differentiation of the inner root sheath [25]. Therefore, *MYC* dysregulation may be associated with the appearance of hair on the back of children.

### 4.3. Heart Defects

Heart defects are also a common outcome that was not present either in our case or in his mother. Hilger et al., 2013 [12], described an individual with this characteristic linked with the duplication of only three genes (*PLEC*, *EPPK1*, *PARP10*). Even though these genes seem to not have any relationship with cardiac formation, we were able to verify that all individuals but two (those described by Bonaglia et al., 2005 [10] and Sun et al., 2021 [15]), with these genes duplicated also showed a heart defect.

### 4.4. Neurologic Aspects

The main clinical manifestation in our index case (son), and one of the most prominent findings of the literature review, is intellectual deficiency. In these studies, some cases had the brain-specific angiogenesis inhibitor 1 (*BAI1*; also called *ADGRB1* or *B1*) gene in the duplicated region. This is interesting because the breakpoints of our case suggest a disruption of *BAI1*. Changes in the expression of BAI1 protein affect the formation of dendrites, causing its retraction or making them immature and unstable. It also affects neuronal plasticity, due its effect on postsynaptic density 95 (PSD-95) protein expression. The proper function of the system formed by BAI1 and PSD-95 seems to be essential for learning and memory, and this is often altered in neurodevelopmental and neurological disorders, leading to impaired social interaction, communication deficits, and increased repetitive behaviors [26,27]. 

In spite of his mother having only a learning disability, it was noticed that the burden of larger CNVs is greater in patients with more severe developmental phenotypes [28]. In our case, the mother and son seemed to share the same duplication length. However, even though we know that the *BAI* gene is disrupted, due to the resolution of the technique with the distance between oligonucleotides ranging 40.000 base pairs, the extent of alteration cannot be precise in each case. Considering this and adding the complicated meiosis pairing illustrated in Figure 3, it is possible that an error on the chromosome synapsis changed the genetic content, which would justify the variation in their neurological manifestation.

Regarding seizures, the *KCNQ3* gene, which codes the Kv7.3 channel, is a prominent candidate since the abnormal discharge of brain neurons represents the foremost feature associated with epilepsy [29]. To date, there are three *KCNQ3*-related disorders: self-limited familial neonatal epilepsy (SLFNE); self-limited familial infantile epilepsy (SLFIE); and KCNQ3-related neurodevelopmental disorder (NDD). The last can present as developmental and epileptic encephalopathy (DEE), a group of rare and severe epilepsies that commonly begin in infancy or childhood and are associated with aggressive epileptogenic activity during brain maturation and cognitive and neuropsychological stagnation or regression [30]. Usually, in these cases, as in our patient, the brain MRI is normal.

Also, as we could diagnose in our patient, mutations in *KCNQ3* lead to sharp waves over the frontotemporal areas on EEG, and it can be controlled by valproic acid [31]. This kind of sign is typical of temporal lobe epilepsy (TLE), one of the most common and complex forms of epilepsy [29]. Valproic acid seemed to control the epileptic episodes of our case very well. Intriguingly, although Bonaglia et al., 2005 [10], and Rezek et al., 2014 [3], also reported patients with epilepsy, none of their duplication range comprises the *KCNQ3* gene. In addition, others with alterations that cover this gene, including our patient’s mother, do not report epilepsy or seizures. This fact may highlight the importance of genomic configuration in gene expression, as explored above.

### 4.5. Miscarriages

In addition, regarding the pedigree chart, inversions are known to increase individuals’ predisposition to other rearrangements [20], and the number of miscarriages seen among his grandfather’s siblings may be a great indication that this change comes from past generations. The meiotic rearrangements from eventual family members with invdup may be more aberrant and lead to life-incompatible karyotypes, due to a complicated meiosis pairing, as illustrated in Figure 3. We have no access to the biological material from other family members, but it is possible that, as occurs in Recombinant 8 syndrome, another chromosomal alteration runs in this family, which predisposes individuals to our patient’s outcome.

In conclusion, our case may provide relevant information about chromosome 8 pure duplication. Because it is small, we can better visualize the consequences of gene dosage in a delimited area. However, as Hamlet once professed: “There are more things in Heaven and Earth than are dreamt of in your philosophy” [32]; thus, the inverted genomic configuration may be, at least in part, responsible for alleviating some of the symptoms of our patient and his mother, as well as, have an impact of its manifestation on each of them. Even though the relationship between genomic organization and gene expression remains unclear, it seems to be an important piece of the variable expressivity in this case, illustrating the importance of new reports and studies about this subject.

## Figures and Tables

**Table 1:** Comparison of phenotypic features among studies.

		Present case		Ergun et al., 2004 [9]	Bonaglia et al., 2005 [10]	Wheeler, 2010 [11]	Concolino et al., 2012 [2]	Hilger et al., 2013 [12]	Rezek et al., 2014 [3]	Farcas et al., 2019 [13]	Lin et al., 2021 [14]		Sun et al., 2021 [15]	**Total of cases with phenotype**
		Index case	Mother								Case 1	Case 2		
		q24.13-q24.3	q24.13-q24.3	q22.3~q23-qter.	8q24.3	8q23.3-q24.1	8q22.2-q24.2	8q24.3	q23.3–q24.1	q24.12-q24.3	q24.12q24.3	q24.12q24.3	q24.3	
		invdup(8)	invdup(8)	t(1;8)	invdup(8)	dup(8)	dup(8)	dup(8)	t(8;22)	dup del(8)	dup (8) x6~7	dup (8) x6~7	t(8;21)	
		GRCh38/hg38	GRCh38/hg38	NA	NA	NA	NCBI36/hg18	NCBI36/hg18	GRCh37/hg19	GRCh37/hg19	GRCh37/hg19	GRCh37/hg19	NA	
		125385074–142496610	125385074–142496610	NA	NA	116147673–129419458	100338614–145464363	145012210–145132100	116147673–129419458	119488147–145984903	119328435–146295771	119261902–146295771	140238896–14629577	
	Inheritance	Maternal	Unknown	de novo	de novo	Unknown	de novo	de novo	Maternal #	de novo	de novo	de novo	Paternal ##	
	Growth delay	-	-	+	+	-	+	NA	NA	NA	NA	NA	+	4
	Low weight	-	-	+	+	-	+	NA	NA	NA	NA	NA	+	4
	Short stature	-	-	-	+	-	+	NA	NA	NA	NA	NA	+	3
Head and neck	Microcephaly	+	-	+	-	+	-	NA	NA	+	NA	NA	-	4
	Prominent fronthead	-	-	+	NA	NA	+	NA	+	+	NA	NA	-	4
Facial features	Hypertelorism	-	-	+	NA	+	+	NA	+	+	NA	NA	+	6
	Palpebral slant alteration	-	Down	Up	NA	Down	NA	NA	NA	NA	NA	NA	Down	4
	Philtrum alterations	-	-	NA	Short	Short	Long	NA	NA	NA	NA	NA	Long or short *	4
	Microretrognathia	-	-	NA	NA	+	+	NA	NA	-	NA	NA	+	3
	Cleft lip/palate	-	+	-	NA	NA	NA	NA	+	+	NA	NA	-	3
	High palate	-	-	NA	+	NA	+	NA	NA	+	NA	NA	-	3
	Nasal bridge alteration	-	-	Depressed	NA	NA	Large	NA	Shallow	Broad	NA	NA	Broad	5
	Large nose	-	-	NA	+	NA	NA	NA	+	NA	NA	NA	+	3
	Low-set ears	-	-	+	NA	+	+	NA	+	+	NA	NA	-	5
	Large ears	-	-	NA	+	NA	NA	NA	NA	NA	NA	NA	+	2
	Protruding ears	-	-	NA	NA	+	-	NA	NA	-	NA	NA	+	2
Visual anomalies		-	-	NA	NA	+	+	NA	NA	-	NA	NA	+	3
Limb anomalies		-	-	NA	+	+	+	NA	NA	+	NA	NA	-	4
Skin anomalies	Hypertrichosis	+	-	NA	NA	+	-	NA	NA	NA	NA	NA	NA	2
Neurologic aspects	Hypotonia	-	-	NA	NA	+	-	NA	NA	-	NA	NA	-	1
	Seizures/epilepsy	+	-	NA	+	NA	-	NA	+	NA	NA	NA	-	3
	Learning disability	+	+	NA	NA	NA	NA	NA	NA	NA	NA	NA	NA	2
	Intelectual deficiency	+	-	+	+	+	+	NA	+	Unknown	NA	NA	+	7
	Motor delay	+	-	+	+	-	-	NA	+	NA	NA	NA	-	4
	Hearing loss	-	-	NA	NA	+	-	NA	NA	-	NA	NA	-	1
Other systems	Heart defects	-	-	+	NA	+	+	+	NA	+	+	+	-	7
	Skeletal abnormalities	-	-	-	Malar hypoplasia	NA	Flat occiput	Butterfly vertebra	NA	NA	NA	NA	Scoliosis	4
	Cryptorchidism	-	female	NA	female	female	+	+	female	female	NA	NA	-	2
	Anal imperforation	-	-	NA	NA	NA	NA	+	NA	+	NA	NA	+	3
	Hernia	-	-	Inguinal	NA	NA	Umbilical	NA	NA	NA	NA	NA	NA	2

* Text refers as short philtrum, but table as long; # Due to non balanced segregation of a translocation [46,XX,t(8;22)(p21.1;q22.3)]; ## Due to non balanced segregation of a translocation [46,XY,t(8;21)(q24.3;q22.3)].

## Data Availability

The original contributions presented in the study are included in the article/supplementary material, further inquiries can be directed to the corresponding author.

## Supplementary Materials

The following supporting information can be downloaded at: https://www.mdpi.com/article/10.3390/genes15070910/s1, Table S1: Comparison of phenotypic features among studies. Table S2: Gene annotation [2,3,9,10,11,12,13,14,15].
